# Ongoing Laryngeal Stenosis: Conservative Management and Alternatives to Tracheostomy

**DOI:** 10.3389/fped.2020.00161

**Published:** 2020-04-15

**Authors:** Cláudia Schweiger, Denise Manica

**Affiliations:** ^1^Otolaryngology Department, Hospital de Clínicas de Porto Alegre, Porto Alegre, Brazil; ^2^Programa de Pós-Graduação em Saúde da Criança e do Adolescente, Universidade Federal do Rio Grande do Sul, Porto Alegre, Brazil

**Keywords:** airway stenosis, larynx, acute lesions, intubation, laryngoplasty

## Abstract

**Background:** Following tracheal intubation, some children may develop stridor, which is an indication of an obstructive lesion in the airway, such as an ongoing laryngeal stenosis (LS). This review focuses on evaluation of stridor and possible endoscopic predictors of progression to LS and, once post-intubation acute lesions are established, therapeutic choices to manage this disorder in avoidance of tracheostomy. Tracheostomy, due to its inherent increased morbidity, mortality and influence on social stigma, should be viewed only as a last resort. In this article, available conservative and alternative therapies for ongoing LS are thoroughly reviewed.

**Methods:** A systematic review concerning randomized clinical trials and prospective studies on treatment modalities for LS was performed. A search strategy was developed for MEDLINE comprising terms related to disease, intervention and population. Title and abstract from captured references were peer-reviewed for eligibility. Selected studies full-texts were peer-reviewed and the results were compiled in a structured and narrative review. Stridor evaluation and post-extubation acute lesion classification were studied. Treatments such as balloon dilation, rigid dilation, corticosteroid-coated small tube intubation, and corticosteroid nebulization were described and evidence supporting their usage was discussed.

## Introduction

Laryngeal stenosis occurs mainly as a consequence of endotracheal intubation. Endotracheal tube induced mucosal injury is a well-recognized etiopathogenic factor leading to stenosis but contributing factors are still a matter of debate. Among these, sedation level (1) and length of intubation (2) have already been demonstrated as definite risk factors.

Definition of acute lesions comprises those identified in <30 days since extubation (3, 4). Moderate to severe lesions pose the highest probability of progressing to chronic stenosis. Recently a classification for acute lesions was proposed with high sensitivity and specificity for the development of laryngeal stenosis. This classification is showed in Table 1 (5).

**Table 1:** Classification of acute laryngeal Injuries (CALI) as mild, moderate, or severe, according to anatomical location and type of injury.

	**Group 1**	**Group 2**	
	**Mild**	**Moderate**	**Severe**
Supraglottis	■ Edema ■ Hyperemia		
Glottis	■ Edema ■ Hyperemia	■ Uni- or bilateral ulceration ■ Arytenoid GT	■ Inter-arytenoid ulceration
			■ Inter-arytenoid GT ■ Immobility
Subglottis	■ Edema ■ Hyperemia	■ Partial ulceration (<360°)	■ Complete ulceration (3,600)
			■ GT

*GT, granulation tissue*.

Endoscopic treatment of acute lesions may avoid eventual tracheostomy and need for open surgery; or result in a less obstructive lesion that may present a better probability of success for a following intervention.

A range of different endoscopic approaches can be used, both isolated or combined. The use of endoscopic treatment is resurging and this can be attributed to the availability of new and more sophisticated endoscopic instrumentation and the adjunctive use of new pharmaceuticals (6). Besides, endoscopic treatment results in a shorter operative time, decreased length of hospitalization, avoidance of external incisions, and less emotional and financial burden on the family.

Despite important advances in the management of subglottic stenosis over the last decades, its treatment remains complex and challenging. The aim of this systematic review is to identify current available endoscopic approaches for this disease and evaluate their success rates.

## Methods

### Information Sources and Search Strategy

We have performed a comprehensive systematic review in order to identify all reports concerning ongoing laryngeal stenosis and therapeutic strategies. Search strategy was developed for MEDLINE and comprised terms defining laryngeal stenosis and related disorders, and available therapies. Annals from major international meetings in otolaryngology and pediatric airway surgery were also consulted. We have also hand searched references from all retrieved publications and prior systematic reviews. The search strategy for MEDLINE is available in Table 2. Further search strategies followed the same structure.

**Table 2:** Search strategy for MEDLINE (accessed: 07/07/2019).

**Search term**	**Number of entries**
**#1:** (“Laryngostenosis”[Mesh] OR “laryngeal stenosis”[tw] OR “subglottic stenosis”[tw] OR “laryngeal stenosis”[All fields] OR “subglottic stenosis” [All fields]) OR (“stridor” AND “intubation” [All fields]) OR (“acute laryngeal stenosis” [All fields])	4,192
**#2:** (Infant[MeSH] OR Infant* OR infancy OR Newborn* OR Baby* OR Babies OR Neonat* OR Preterm* OR Prematur* OR Postmatur* OR Child[MeSH] OR Child* OR Schoolchild* OR School age* OR Preschool* OR Kid or kids OR Toddler* OR Adolescent[MeSH] OR Adoles* OR Teen* OR Boy* OR Girl* OR Minors[MeSH] OR Minors* OR Puberty[MeSH] OR Pubert* OR Pubescen* OR Prepubescen* OR Pediatrics[MeSH] OR Pediatric* OR Pediatric* OR Peadiatric* OR Schools[MeSH] OR Nursery school* OR Kindergar* OR Primary school* OR Secondary school* OR Elementary school* OR High school* OR Highschool*)	4,582,304
**#3:** (corticosteroid[tw] OR steroid[tw] OR balloon*[tw] OR dilat*[tw] OR rigid*[tw] OR “Mitomycin”[Mesh] OR “1a-docosahexaenoyl mitomycin C” [Supplementary Concept] OR “mitomycin C-N (2)-deoxyguanosine adduct” [Supplementary Concept] OR “mitomycin C-DNA adduct” [Supplementary Concept] OR “mitomycin C-immunoglobulin M antibody conjugate” [Supplementary Concept] OR “mitomycin C-dextran” [Supplementary Concept] OR “mitomycin C-anti-alpha-fetoprotein antibody conjugate” [Supplementary Concept] OR mitomycin[tw] OR “cricoid split”[tw])	543,280
**#1** AND **#2** AND **#3**	428

Duplicates were excluded before proceeding to study selection. All titles and abstracts retrieved were screened independently by two researchers. Full-text articles also had its eligibility evaluated by two independent researchers. The last date of the search was July 7th, 2019. We have followed Preferred Reporting Items for Systematic Reviews and Meta-Analyses (PRISMA) statement for conducting this study and reporting our results (7, 8).

### Eligibility Criteria

#### Inclusion Criteria

We have included all references concerning randomized clinical trials (RCT), phase I and II prospective studies, case series and case reports, involving neonatal (0–28 days old) and pediatric (29 days to 18 years) patients, and reporting results on pharmacologic and endoscopic treatments as primary approaches for ongoing laryngeal stenosis. Only reports in English, Portuguese, Spanish, Italian and German were eligible.

#### Exclusion Criteria

We have not included other types of publications such as reviews, editorials, letters to the editor, guidelines, study protocols or position papers. All open surgical approaches (even if endoscopic procedures were performed as adjunctive treatment) were excluded. Also, studies pertaining to population samples concerning adult patients (both exclusively or alongside pediatric patients) were excluded. Congenital stenosis or those acquired by other causes not associated with endotracheal intubation were excluded because they do not share the same pathologic mechanisms.

### Study Selection and Review

Two reviewers participated in the screening and full-text evaluation. All abstracts screened and articles selected were reviewed by the same two reviewers, while a third reviewer would intervene if there was any discordance over eligibility. Reports were classified based on their main focus, whether diagnosis, prognosis, treatment and complications. All reviews were proceeded independently, and final drafts from both reviewers were eventually compiled into one final review article.

## Results

### Search Results

**Figure 1:**
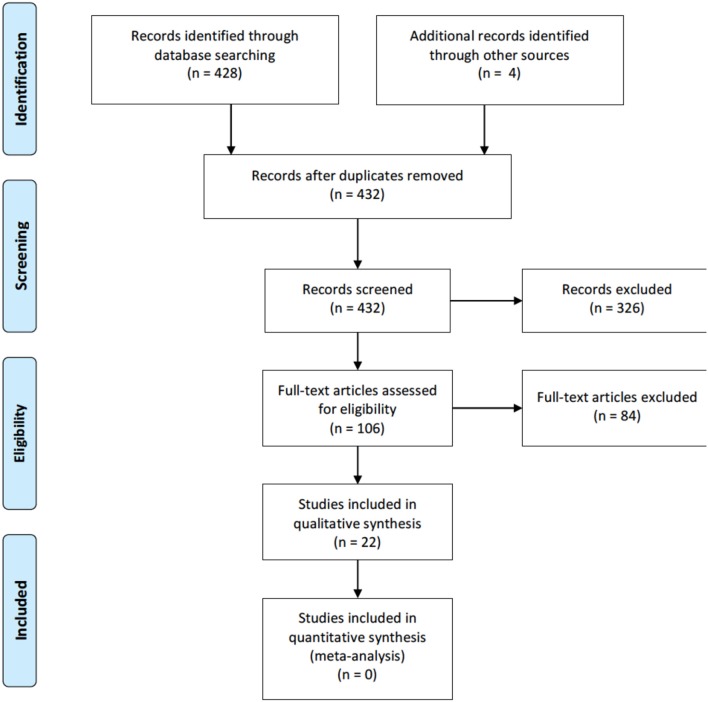
PRISMA flow diagram.

The initial search resulted in 432 references. After excluding duplicates and articles not concerning study's population and/or intervention, 106 were eventually assessed as full text. Finally, 22 articles fulfilled eligibility criteria and were included in qualitative synthesis. No quantitative meta-analysis of data was possible due to heterogeneity of interventions and scarcity of comparative trials. Studies content was summarized below. PRISMA flow diagram is depicted in Figure 1.

### Elective Endotracheal Intubation

Hoeve et al. described 23 patients with a mean age of 37 days who presented extubation failure due to acute laryngeal lesion. They were re-intubated with a “loose fit” tube (inferior in diameter to what would be recommended concerning patient age, permitting leakage of air at the end of an insufflation) for a period of 17 days. Only one of these patients progressed to tracheostomy. When the airway finding was edema or superficial lesions, mean time for intubation was only 8 days and more than half of these patients were extubated within 3 days. When ulceration and edema was found, the mean intubation time was 13 days. However, when granulation was present, intubation longed for weeks afterwards (9).

Graham reported 10 newborn patients undergoing a protocol of 2 weeks of intubation after extubation failure due to acute lesion. Six patients were successfully extubated; two were tracheostomized and decannulated afterwards, with no need for further surgical procedures; one underwent tracheal reconstruction; and the other tracheostomized patient died due to other causes (10).

Monnier recommends that, when dealing with soft-tissue stenosis without mucosal necrosis, a re-intubation should be performed with a one-size smaller endotracheal tube with topical application of an endolaryngeal plug of gentamycin-corticosteroid ointment. Most of these patients can be extubated after a mean re-intubation period of 2–4 days (11).

### Endoscopic Balloon Dilation (EBD)

Balloon dilation was first described in 1984 as a method to treat tracheal and bronchial stenosis (12) Subsequently, other case reports were published (13–15). All of them, however, applied balloon dilation for patients with chronic stenoses. The analysis of balloon dilation results for chronic and acute stenoses as separate diseases was only recently attempted.

Compared to previous methods of dilation such as bougies and endotracheal tubes, this new technique has the theoretical advantage of exerting radial pressure on the airway, which projects the stenosis away from the center of the airway, reducing the incidence of shear-related trauma of the epithelium that may occur with rigid dilation (16). Balloon dilation has been studied in several case series since 1991, but its indications, safety and efficacy were still under debate.

The literature search identified 24 abstracts about balloon dilation. After review of the full-length articles, 14 were excluded, due to its use for chronic stenoses (15, 17–20), due to its lack of differentiation between acute or chronic stenosis (16, 19, 21–27), and/or because the paper did not differentiate between rigid or balloon dilations (27, 28). The 10 remaining papers were selected for this review (3, 29–37).

#### Technique

Balloon dilation is performed under general anesthesia in all articles, using a high pressure, non-compliant balloon catheter. The technique has a slight variability in the studies.

Some authors used the INSPIRA AIRTM balloon (Acclarent Inc., CA, U.S.A.) (33, 37). Other authors do not mention the brands of their balloons and/or use many different brands (3, 29–32, 34–36). The balloon size was selected according to the ideal subglottic diameter for the patient's age (3, 29, 32–34, 36, 37). An inflation/deflation handle mounted with a syringe and gauge assembly designed to monitor and maintain the pressure was used by all authors (29–38). The balloon was inflated to rated burst pressure by some authors (33, 36, 37). Other authors specifically mentioned pressures between 2 and 15 atm, (3) 2 atm, (29, 31, 34), 4 atm, (32), 7 atm (30). The balloon was maintained inflated for 30–60 s or until the patient's oxygen saturation level dropped below 90–92% (3, 29–37). Some authors repeated the procedure 2–3 times during each session (31–37).

#### Outcomes of Balloon Dilation

The 10 case reports and series (nine retrospectives, one prospective), published between 2007 and 2017, included 109 pediatric patients who underwent 1–6 dilations each, with an average 1.8 dilations per patient. Follow-up ranged from 5 days to 7 years (3, 29–37). Included studies are summarized on Table 3.

**Table 3:** Balloon dilation: case reports and case series (*n* = 109).

**Author, year. Retrospective X prospective**	**Number of patients. Age**	**Description of the type of SGS. Grade of SGS**	**Number of dilations. Mean number of dilations per patient**	**Adjuvant treatment**	**Success rate. Follow-up length**
Durden and Sobol (29). Retrospective case series	Four patients. 3–7 mo	Soft. Grades 2–3	1–2 dilations per patient. Mean: 1.2	Topical steroid applied + intubation for 24–48h + systemic steroid	75% success.^a^ Mean of 3.5 months of follow-up
Rossetti et al. (30). Retrospective case report	One patient. 10 mo	Acute. Grade 3	1 dilation.	None	100% success. 5 days of follow-up
Schweiger et al. (31). Prospective case series	Eight patients. 2–14 mo	Acute (<60 days after intubation, with GT). Grades 1–3	1–2 dilations per patient. Mean: 1.37 dilations per patient	Systemic steroids	100% success. 6 months of follow-up
Collins et al. (32). Retrospective case series	Two patients.^b^ 1 mo (both)	Thin, circumferential SGS. Grade 3	2 dilations. Mean: 1	Topical Mitomycin (1 patient) and topical triamcinolone (1 patient)	100% success. 1–23 months of follow-up
Whigham et al. (33). Retrospective case series	Nine patients.^c^ 1–31 mo (mean = 10.8 mo)	Soft. Grade 2–3	14 dilations. Mean: 1.55 per patient	None	66% success. 12–24 months of follow-up
Filiz and Ulualp (34). Retrospective case series	Three patients. 14 weeks-1 yo	Acute (5 days to 6 weeks after extubation). Grades 2–3	3–4 dilations per patient. Mean: 3.33 per patient	Systemic steroids	100% success. 14–21 months of follow-up
Avelino et al. (3). Retrospective	17 patients.^d^ Mean: 4.2 mo	Acute (<30 days after extubation). Grades 1–3	Mean: 2 dilations per patient.	Oral prednisolone + steroid nebulization	100% success. 3–15 months of follow-up (mean 7.8 months)
Ozturk et al. (35). Retrospective case report	One patient. 23 days-old	Acute. Grade 3	2 dilations.		100% success. 8 months of follow-up
Alshammari et al. (37). Retrospective	45 patients.^e^ 1 mo−15 yo	40 soft, 5 mature. Grades 1–3	1–6 dilations per patient. Mean: 2	1–2 ml Kenalog	82.3% success. 1 year of follow-up
Chen et al. (36). Retrospective	19 patients.^f^ 0.3–144 mo (mean = 4 mo)	Acute. Grades 2–4.	1–8 dilations per patient. Mean: 2.6 per patient		100% success. 0.5–7 years of follow-up

*mo, months-old; yo, years-old; SGS, subglottic stenosis; GT, granulation tissue*.

a *One patient in the study needed a tracheostomy*.

b *Only patients number two and four were included in this analysis (not clearly described in the paper if the other three patients presented acute or chronic lesions)*.

c *Only nine patients with soft stenosis and primary balloon dilation (excluded those who underwent adjuvant balloon dilation and those who had firm lesions)*.

d *Only patients with acute lesions were included in this analysis*.

e *Authors described 40 patients with acute lesions and five with chronic ones. They do not separate outcomes of acute and chronic lesions, but since chronic ones accounted for only 12.5% of their patients, it was decided to include all their patients in this analysis*.

f *Only patients with acute SGS and who underwent balloon dilation were included in the analysis. Of the 22 patients with acute lesions, three were excluded because they were submitted to balloon dilation + endoscopic anterior cricoid split*.

Success was defined as an improvement of symptoms, decrease in Myer-Cotton level of stenosis, decannulation of previous tracheostomy, or avoidance of reconstructive surgeries and tracheostomy in all studies. Success rate ranged from 66 to 100%, with a total of 97 of the 109 patients (88.99%) reaching a successful outcome.

None of the studies described complications of the procedure. Avelino et al. described that four patients presented dysphagia in the post-operative period, but they do not specify whether these patients had previous acute or chronic SGS (3).

#### Balloon Adjunctive Treatments

##### Mitomycin-C

Mitomycin-C is an antibiotic produced by *Strepnomyces caespitosus* exhibiting antiproliferative and antineoplastic properties. It inhibits fibroblast proliferation and synthesis of extracellular matrix proteins, and thereby modulates wound healing and scarring.

Ortiz et al. described 16 children presenting SGS managed with balloon dilation and topical application of mitomycin solution 1 mg/mL in the dilated area for 1 min, reporting a 100% success rate. Authors describe that laryngoscopy was performed as soon as symptoms appeared, but do not specify the time interval between extubation and examination. Mean number of dilation procedures was 2.5, and the number of sessions was proportional to the grade of stenosis (39).

##### Endoscopic Anterior Cricoid Split (EACS)

Open surgical approach was first described by Cotton as an alternative to performing tracheotomy in premature infants with prolonged intubation. As the aim of the present review is conservative treatments, only EACS is reviewed.

Mirabile et al. (40) described for the first time EBD-associated EACS. They reported five acquired stenosis patients without tracheostomy in whom a 100% success rate was attained. After this first study, Chen et al. (36) described three children presenting acute SGS undergoing EBD + EACS with a success rate of 66.7%. Similarly, Horn et al. (41) described success with the technique in two out of three children presenting extubation failure. Carr et al. (42) described a 100% success rate in five intubated children with extubation failure, using this same approach.

### Rigid Dilation

The literature search identified 14 abstracts about rigid dilation. After review of the full-length articles, only one of those studies clearly separated acute and chronic lesion groups, which was eventually included (4).

Dilations were performed with increasing diameter endotracheal tubes, depending on the patient's age. Dilation was started with Silastic (a portmanteau of “silicone”and “plastic”) bougies when stenosis prevented the introduction of a 2.5 mm endotracheal tubes. In 12 patients, adjuvant treatment was administered locally, but authors did not separate groups (acute or chronic) for this intervention. Success rate was 96.4% (27/28) and researchers determined that acute stenosis was a predictor of success, while chronic stenosis showed a success rate of 57.1%.

### CO_2_ Laser and Coblation

Studies concerning different laser and coblation techniques in laryngeal stenosis deal mainly with chronic stenosis (43–46). Regarding acute lesions, a study by Koufman et al. (47) was found describing five non-tracheostomized patients undergoing stenosis treatment with carbon dioxide (CO_2_) surgical laser. Two of them needed tracheostomy, but eventually were decannulated. Dilation and steroid injection were adjunctive measures. Bollig and Gov-Ari (48) described a 9-months-old girl successfully managed with bipolar radiofrequency plasma ablation (coblation) after prior multiple endoscopic balloon dilations.

### Microdebrider

Rees et al. (49) described one case of subglottic granulation treated with microdebrider. This was the sole publication reporting the use of this technique in acute lesions.

## Discussion

In 1984, Cotton stated that endoscopic techniques were effective in the early phases of wound healing, when the scar tissue is soft and pliable (50). Since then, many techniques have been described, with variable rates of success. Recent technological advances have facilitated endoscopic approaches that promise fewer wound complications, decreased postoperative pain, shortened hospital stays, and no external scarring (40). The success of endoscopic techniques for acute stenosis is defined by the long-term resolution of stenosis and the avoidance of open surgery such as laryngotracheal reconstruction.

Rigid dilations have been used for a long time, with highly variable and essentially disappointing results (51, 52). Rigid dilation studies found in this review do not report clear inclusion criteria especially concerning mature or in evolution stenosis status. Also, miscellaneous etiologies and different age groups were enrolled. Furthermore, reported dilatation techniques and ancillary treatments are heterogeneous. It is known that although this technique is effective, significant sheer forces are generated across the area of stenosis, and ongoing serial dilatations are often required. Studies on rigid dilation showed the need of a high number of repeated dilations but again did not segregate chronic from acute lesions. The only one found stratifying this information reported a mean number of procedures of two and a success rate of 96.4%.

Balloon dilation emerged as a promising alternative to those dilations with bougies, bronchoscopes and endotracheal tubes. It was initially designed for endovascular interventions and later adapted for use in tracheobronchial stenoses by interventional radiologists and pediatric surgeons (12, 13). The absence of shearing forces minimizes subglottic trauma, both at the mucosal level and in deeper planes, thereby decreasing the tendency to re-stenosis. As with conventional dilation techniques, balloon dilation is more likely to succeed in the presence of immature scar tissue (15).

While the number of reports on balloon dilation is still currently small, encouraging reports of this simple technique suggest that balloon dilation is probably the single most promising treatment for acute subglottic stenosis.

There is very little information available in the literature to guide balloon sizing, inflation pressure and length of inflation time. Currently, surgeons often use their own breadth of experience to guide the parameters used in the procedure. Excessively high inflation pressures or big balloon sizes can damage or rupture the airway, and inadequately low pressures or small balloon sizes can reduce the effectiveness of procedures and result in the requirement for even more procedures.

Lee et al. (28) was excluded of the balloon dilation analysis, but interestingly its results showed that, independently of the kind of therapy (bougination, incision using cold knife or laser, and balloon dilation), the success rate for acute SGS (within 30 days after extubation) is 88.8%, compared to 9.09% for chronic stenosis (more than 30 days after extubation). Their success rate sharply equals the success rate of this systematic review (88.99%). In the discussion, authors mention that the success rate is probably related to the SGS grade and nature of the scar tissue, since fine membranous scars and acute membranous type of SGS (immature scar tissue) can be expanded more easily than total cartilaginous fibrosis (mature scar tissue).

However, since the outcome of the dilation was not 100% in many studies, adjuvant therapies, such as steroid local injection, systemic steroids have been added to the therapeutic options in some cases. As we could notice in this systematic review, evidences about these adjuvant therapies are scarce. Also concerning other approaches such as CO_2_ laser, coblation and microdebridor, further studies are necessary in acute lesions to settle debate over those specific treatments.

The establishment of endoscopic management protocols is complex due to technique variability and multiplicity among different institutions and patient's individual characteristics. Success of endoscopic treatment is probably also significantly influenced by the skill and ability of the collaborative staff, especially anesthesiologists and neonatal/pediatric critical care specialists. Table 4 summarizes success rates from therapeutic approaches discussed in this review.

**Table 4:** Comparison of therapeutic approaches success rates.

**Therapeutic option in acute laryngeal lesions**	Success rates^*^
Elective endotracheal intubation	60 (10)−95.6%(9)
EBD	66 (33)−100% (36)
EBD + mitomycin-C	100% (39)
EBD + EACS	66.7 (36)−100% (40)
Rigid dilation	96.4% (4)
CO_2_ laser	60% (47)
Coblation	1 successful case (48)
Microdebrider	1 successful case (49)

*EBD, Endoscopic Balloon Dilation; EACS, Endoscopic Anterior Cricoid Split*.

* *Included studies did not report on complications rates from therapeutic approaches*.

As far as authors are concerned, if the acute lesion is restricted to subglottis, EBD is the best option. In the postoperative period, steroid nebulization and proton pump inhibitor use has become a promising adjuvant treatment. Review laryngoscopy around the seventh postoperative day should always be performed, or even earlier if symptoms become noticeable. Procedure may be repeated many times, as long as the subglottic lumen is improving with each dilation. If the lumen is still not good enough after the second dilation, adjuvant treatment as topic steroids are advisable. Authors do not recommend more than 4 dilations–if the patient would require more than 4 dilations, it seems reasonable to change the therapeutic approach. When there is an associated glottic lesion or widespread airway edema or granulation tissue, reintubation with a one-or two-size smaller endotracheal tube with topical application of a steroid ointment for 48–72 h seems to be an attractive approach. Soon after extubation, we also recommend the use of inhaled corticosteroid and proton pump inhibitor.

Future studies should examine the effects of different balloon diameters and pressures, the role of adjuvant therapies such as steroid local injections and systemic steroids, and also the difference between rigid and balloon dilation. There are currently no evidence-based guidelines for the treatment of post-intubation acute laryngeal lesions, and the clinical experience of each team often guides the therapeutic approach.

## Data Availability Statement

The raw data supporting the conclusions of this article will be made available by the authors, without undue reservation, to any qualified researcher.

## Author Contributions

CS and DM participated in study design, search strategy elaboration, peer-review, narrative review, manuscript writing, and final revision.

### Conflict of Interest

The authors declare that the research was conducted in the absence of any commercial or financial relationships that could be construed as a potential conflict of interest.
